# Biological importance of podoplanin expression in chorionic villous stromal cells and its relationship to placental pathologies

**DOI:** 10.1038/s41598-019-50652-9

**Published:** 2019-10-02

**Authors:** Nilufer Onak Kandemir, Figen Barut, Aykut Barut, İsmail Eren Birol, Banu Dogan Gun, Sukru Oguz Ozdamar

**Affiliations:** 1 Department of Pathology, Ankara Ataturk Training and Research Hospital, Ankara, 06310 Turkey; 2 Department of Pathology, Faculty of Medicine, Bulent Ecevit University, Zonguldak, 67600 Turkey; 3 Department of Obstetrics and Gynecology, Faculty of Medicine, Bulent Ecevit University, Zonguldak, 67600 Turkey

**Keywords:** Cell adhesion, Immunopathogenesis

## Abstract

Podoplanin, a reliable marker of lymphatic endothelium, is a mucin-type transmembrane protein. Although the human placenta is devoid of a lymphatic system, chorionic villous stromal (CVS) cells express podoplanin. In this study, the pattern of podoplanin expression in normal and pathological placental tissues and the biological role of podoplanin were investigated. In total, 198 placental tissues belonging to 184 patients, seen at the Department of Pathology of Bulent Ecevit University Education and Research Hospital, Zonguldak, Turkey, were evaluated histopathologically and determined to meet the study criteria. The tissues were assigned to control, cisternal placental disorders, inflammation and hypoxic-ischemic pathology groups. Podoplanin expression in CVS cells was graded from 0 to 3 depending on the staining intensity, as determined by an immunohistochemical evaluation of chorionic villi in the most intensively stained tissue region. Podoplanin levels in control CVS cells increased in parallel with placental maturation, whereas in molar pregnancies podoplanin expression was lower than in control tissues. In the acute placental inflammation group, podoplanin immunoreactivity was similar to that in the control group, whereas in the preeclampsia group, podoplanin expression was higher than in all other groups. Our study showed an increase in podoplanin expression in CVS cells during pregnancy. In preeclamptic patients, the increase in podoplanin expression may be a response to hypoxic-ischemic conditions, whereas in molar pregnancies the decrease in podoplanin levels may cause villous swelling by disrupting intercellular fluid homeostasis.

## Introduction

Podoplanin is a mucin-type transmembrane protein composed of 162 amino acid residues. Podoplanin’s extracellular domain contains four platelet-aggregating (PLAG) domain repeats, which interact with C-type lectin 2 (CLEC-2) receptors on the surface of platelets. CCL21, Galectin 8, CD9, CD44, and MMP14 are also ligands of podoplanin^1,2^ The intracellular sequence contains basic amino acids and serine residues, the association of which with ezrin-radixin-moesin (ERM) proteins directs RhoA GTPases to reorganize the actin cytoskeleton. Podoplanin-mediated actin remodeling affects cellular motion^3,4^. As a result, podoplanin plays an important role in a variety of physiological and pathological (e.g., inflammation, thrombus formation, and cancer progression) processes, as well as in cellular adhesion, migration, and chemotaxis. Podoplanin expression is upregulated in both epithelial and mesenchymal cells during ischemia-hypoxia, inflammation, and cancer. Various agents, including growth factors (e.g., VEGF-C and TGF-β1), cytokines (e.g., TNF-α, IFN-γ, IL-1, and IL6), fibronectin, and lipopolysaccharides, increase podoplanin expression^1–4^.

Podoplanin was first described by Dobbs *et al*.^5^ in type 1 pneumocytes, but it has since been identified in a large number of cell types originating from different germinal layers. Among the many physiological and pathological processes in humans in which podoplanin plays an important role are vasculogenesis and lymphangiogenesis^1–4^. Placental tissue is one of the few immunologically protected regions that lacks a lymphatic system. However, podoplanin is present in the stromal cells of the chorionic villous, where in chorionic villous stromal (CVS) cells its expression pattern is similar to that in immature lymphatic channels^6,7^.

While the function of podoplanin in different human tissues has been investigated, the biological significance of podoplanin in placental tissue is unclear. The few studies that investigated podoplanin expression in normal and pathological placental tissues, and the role of the protein in placental pathologies, yielded contradictory results. A PUBMED search using the keywords placenta-podoplanin-D2-40 identified 12 articles, the first of which was published in 2008^6–17^. Most of these studies investigated the presence of a mature lymphatic system in the placenta; their findings are summarized below.

Bellini *et al*.^6^ found no evidence of mature lymphatic vessels in chorionic villi, but noted that stromal cells were positive for podoplanin. The authors argued that podoplanin-expressing stromal cells function as primitive lymphatic channels. Wang *et al*.^7^ suggested that podoplanin plays an important role in fetal angiogenesis during placental development, and that a decrease in podoplanin activity leads to gestational diseases associated with defective angiogenesis. In the study of Volchek *et al*.^11^, non-decidualized gestational endometrium was shown to be rich in lymphatic vascular structures, which regressed with increasing decidualization. Also, the evaluated decidua did not contain lymphatic vessels. Lee *et al*.^9^ reported the presence, in the umbilical cord and chorionic villi, of a podoplanin-positive primovascular system (PVS) whose placental functions include immunomodulation, tissue regeneration, and stemcell modulation. Castro *et al*.^10^ proposed that lymphatic vascular changes constitute part of the decidual vascular remodeling that occurs during the gestational period. The impaired lymphangiogenesis detected by Heazell *et al*.^15^ was suggested to play a role in the pathogenesis of placental mesenchymal dysplasia. In addition, a stem cell function was attributed to podoplanin-positive CVS cells. Lundell *et al*.^17^ showed that CVS and maternal decidual stromal cells are the major sources of podoplanin in the human placenta, and that podoplanin-secreting cells in placental tissue play a role in feto-maternal immunological tolerance.

In this study, we investigated the expression pattern of podoplanin in CVS cells in normal and pathological placental tissues using an immunohistochemical method. We aimed to determine the role of podoplanin in normal placental development and its effect on the development of inflammatory, hydropic, and hypoxic-ischemic placental diseases. We also compared our results with those in prior reports.

## Materials and Methods

### Case selection

Between January 2011 and December 2016, 198 placental tissues belonging to 184 patients seen at the Department of Pathology of Bulent Ecevit University Education and Research Hospital, Zonguldak, Turkey, were evaluated histopathologically and determined to meet the study criteria. Insufficient tissue for histomorphological/immunohistochemical evaluation (e.g., abortion materials without adequate chorionic villi, the presence of autolysis/necrosis, structural placental anomalies such as chorangiosis/mesenchymal dysplasia) and tissues from patients without maternal-fetal follow-up data were excluded from the study. All pathological archival materials (NOK and FB, experienced pathology specialists in the field of placental pathology) and medical records (AB, gynecologist) were examined in terms of study eligibility criteria.

### Tissues and histopathological evaluation

Following macroscopic evaluation, the entire curettage material was sampled for histopathological examination. Macroscopic evaluation and sampling of intact placental tissues were performed according to standard pathological procedures^18^. The tissues were fixed in 10% buffered formalin and processed according to standard procedures in a fully automated tissue-processing device. Successively prepared thick sections (4 µm) were stained with hematoxylin and eosin and the monoclonal mouse anti-human podoplanin antibody D2-40 for evaluation by light microscopy according to standard protocols^19–24^. The tissues were divided into eight groups, as follows.

### Control

#### Group 1 (1st trimester, ≤13 weeks of gestation)

46 placental tissues from 42 patients whose pregnancy was terminated for medical or social reasons. Maternal/fetal/placental tissues with pathologies that could significantly affect placental structure and function were not included in this group.

#### Group 2 (2nd trimester, 14–27 weeks)

16 placental tissues from 15 patients whose pregnancy was terminated due to fetal anomalies. Maternal/fetal/placental tissues with pathologies affecting placental morphology were not included in this group.

#### Group 3 (term placenta, 28–40 weeks)

22 term placentas obtained from 20 patients after normal spontaneous vaginal delivery. No maternal-fetal medical pathologies were detected in the tissues obtained from patients in this group. Maternal blood pressure was <140/90 mmHg.

### Hydropic placental pathologies group

#### Group 4 (non-molar hydropic abortion)

31 1st-trimester placentas from 29 patients with histopathologically diagnosed non-molar hydropic abortion according to the criteria in Howat *et al*.^22^ no macroscopic vesicles, trophoblastic hyperplasia, syncytial sprouts, or trophoblasts with an atrophic morphology. No pathology was detected during follow-up after curettage in any patient in this group.

#### Group 5 (molar pregnancy)

31 molar placentas from 29 patients, including 9 cases of complete molar, 3 cases of invasive molar and 17 cases of partial molar pregnancy. P57 immunohistochemical examination, performed on all tissues, maternal follow-up data, and maternal serum β-HCG levels were recorded. The tissues were evaluated immunohistochemically and histopathologically according to the criteria of Howat^22^ and Banet^19^.

### Placental inflammation group

#### Group 6 (acute placental inflammation)

15 placental tissues from 14 patients with acute inflammatory placental lesions, defined as the presence of acute chorioamnionitis and/or funisitis and/or chorionic vasculitis^20^.

### Hypoxic-ischemic placental pathologies group

#### Group 7 (intrauterine growth retardation, IUGR)

25 placentas from 23 patients with IUGR, diagnosed based on obstetric ultrasonographic examinations and fetal measurements. IUGR was defined as a fetal weight or abdominal circumference <10% or 2.5 standard deviations below the normal value for the same gestational week^21^.

#### Group 8 (preeclampsia)

Twelve term placental tissue from 12 patients with preeclampsia, diagnosed based on an arterial blood pressure >140/90 mmHg (at least two different measurements) and proteinuria (>1+ on a dipstick, >300 mg protein in the 24-h urine sample)^23^.

### Immunohistochemical staining and evaluation

Immunohistochemical staining was performed on a fully automatic immunostaining device (BenchMark XT; Ventana Medical Systems Inc., Tucson, AZ, USA) using podoplanin (Clone D2-40, diluted 1:100) and an I-View DAB detection kit (Ventana Medical Systems Inc.). Internal (endometrial blood and lymphatic vascular endothelium) and external (skin-dermal blood and lymphatic vascular endothelium) negative/positive control tissues were included in each staining.

A cytoplasmic and/or membranous reaction was considered positive. In placental tissues, podoplanin immunoreactivity was evaluated and scored in CVS cells. Villous and extravillous trophoblastic cells, Hofbauer cells, decidua, fetal membranes, endometrial glandular epithelium, and endometrial stromal cells were also examined for podoplanin expression, but were not scored. Slides stained with D2-40 were screened at 40× magnification and the most densely stained region was chosen for further evaluation. The chorionic villi showing the most intense spotting in the selected region were scored in terms of staining intensity. For each of the selected chorionic villi, the staining intensity in stromal cells was semiquantitatively graded from 0–3 (0 = no staining, 1+ = mild, 2+ = moderate, 3+ = severe staining). The final score for each case was determined using the “Histo” scoring method [1 × (1 + CVS%) + 2 × (2 + CVS%) + 3 × (3 + CVS%)]^24^.

### Statistical analysis

Statistical analyses were performed using IBM SPSS Statistics for Windows version 21.0 (released 2012; IBM Corp., Armonk, NY). Data are presented as medians (minimum, maximum). The Kruskal-Wallis test was used to determine the significance of differences in H scores among the groups. If a significant difference was found, the Bonferroni Holm test was used for paired comparisons. A *p*-value < 0.05 was considered to indicate statistical significance.

### Ethics statement

Human term placental samples were obtained from unenrolled women with healthy vaginal deliveries who were hospitalized at the Department of Obstetrics and Gynecology, Faculty of Medicine, Bülent Ecevit University, Zonguldak, Turkey. All patients provided written informed consent to participate in the study (if the subject was under 18 years of age, a parent and/or legal guardian consented). The research methods protocol was carried out in accordance with approved guidelines and regulations. The study was performed in accordance with the Declaration of Helsinki, and the privacy of the patients was protected by decoding of data according to the privacy regulations of the Faculty of Medicine, Bülent Ecevit University (Zonguldak, Turkey). This was a retrospective study; thus, approval by the Ethics Committee was not required. No author had access to identifying patient information.

## Results

### Clinical findings

In the present study, which spanned a 5-year period, 198 placental tissue samples of 184 patients who met the inclusion criteria were examined. The mean age of the population was 31.69 ± 7.39 years (17–50 years) and the mean duration of pregnancy was 14.57 ± 8.96 weeks (5–40 weeks). The patients were divided into eight groups based on their clinical and histomorphological characteristics: 41.9% (n = 77) of the population was in the control group (group 1 [first trimester], group 2 [second trimester], and group 3 [term placenta]). Of the patients, 31.4% were in the hydropic placental pathologies group (group 4 [non-molar hydropic abortion] and group 5 [molar pregnancy]); 7.6% in the acute placental inflammation group (group 6); and 19.1% were in the hypoxic-ischemic pathologies groups (group 7 [intrauterine growth retardation, IUGR] and group 8 [preeclampsia]) (Table 1).

**Table 1:** Clinical characteristics of the groups*.

Groups	Age of mother (yr) Mean ± SD, range	Gestational age (wk) Mean ± SD, range	Number of cases (%)	Number of samples (%)
Group 1	30.78 ± 3.36 (17–40)	7.63 ± 1.68 (5–12)	42 (22.8)	46 (23.2)
Group 2	28.85 ± 6.11 (19–39)	17.9 ± 4.18 (13–24)	15 (8.2)	16 (8.1)
Group 3	29.88 ± 7.25 (19–41)	31.06 ± 5.09 (24–38)	20 (10.9)	22 (11.2)
Group 4	32.59 ± 5.61 (24–44)	8.54 ± 0.70 (8–12)	29 (15.7)	31 (15.6)
Group 5	36.17 ± 7.89 (19–50)	10.00 ± 2.11(8–10)	29 (15.7)	31 (15.6)
Group 6	27.40 ± 6.99 (22–40)	29.60 ± 6.38 (28–40)	14 (7.6)	15 (7.6)
Group 7	33.06 ± 7.79 (18–49)	32.43 ± 6.73(28–36)	23 (12.5)	25 (12.6)
Group 8	29.00 ± 30.80 (25–33)	27.60 ± 3.64(24–32)	12 (6.6)	12 (6.1)
Total	**31.69** ± **7.39 (17**–**50)**	**14.57** ± **8.96 (5**–**40)**	**184 (100)**	**198 (100)**

^*^Group 1 (first trimester, ≤13 weeks of gestation), group 2 (second trimester, 14–27 weeks), group 3 (term placenta, 28–40 weeks), group 4 (non-molar hydropic abortion), group 5 (molar pregnancy), group 6 (acute placental inflammation), group 7 (intrauterine growth retardation [IUGR]), and group 8 (preeclampsia). yr, years; wk, week.

### Immunohistochemical findings and statistical results

#### Control group

The control group consisted of first trimester (group 1), second trimester (group 2), and term placenta (group 3) tissues, which exhibited no marked pathology clinically or histomorphologically. The CVS cells of all patients in this group were immunoreactive for podoplanin; however, the density and diffuseness of podoplanin immunoreactivity differed among the patients.

**Figure 1:**
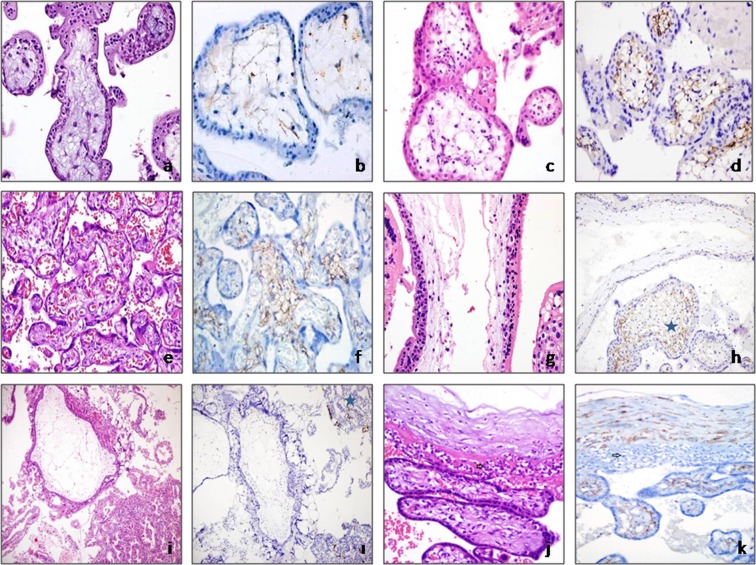
(**a,b**) Group 1: Avascular mesenchymal villi show weak focal podoplanin immunoreactivity (**a**, hematoxylin and eosin [H&E], ×400; **b**, podoplanin, ×400). (**c,d**) Group 2: Significant podoplanin immunoreactivity from the sub-trophoblastic area in secondtrimester placenta, in which fetal vessels are present (**c**, H&E, × 400; **d**, podoplanin, ×400). (**e,f**) Group 3: Diffuse podoplanin immunoreactivity in term placental tissue (**e**, H&E, ×200; **f**, podoplanin, ×200). Group 5: Molar pregnancy groups. (**g,h**) In partial moles, podoplanin loss was observed in cistern-containing villi and podoplanin expression was maintained in normal appearing villi [asterisk] (**g**, H&E, ×630; **h**, podoplanin, ×400). (**i,I**) Total podoplanin loss of hydropic villi in a case of complete molar pregnancy. In the gestational endometrium (internal positive control), the lymphatic endothelium is positive for podoplanin [asterisk] (**i**, H&E, ×200; **i**, podoplanin, ×200). Group 6: In cases of chorioamnionitis, podoplanin immunoreactivity in chorionic villi stromal cells was similar to that in the control group (arrow indicates acute inflammation) (group 3) (**j**, H&E, ×400; **k**, podoplanin, ×400).

In the CVS cells of group 1 (first trimester), podoplanin immunoreactivity was heterogeneous and weak, whereas in groups 2 (second trimester) and 3 (third trimester) it was more intense and homogeneous, and formed a reticular pattern. Hofbauer cells were present in some of the channels formed by podoplanin-positive stromal cells. The lowest levels of podoplanin expression were in tissues from early (<8) gestational weeks (n = 7 cases), in which fetal vascularization had not yet occurred, as podoplanin in CVS cells increased as the gestational period progressed (Fig. 1a–f). The difference in podoplanin expression score between group 1 and groups 2 and 3 was significant (group 1 versus group 2, p < 0.001; group 1 versus group 3, p < 0.001; group 2 versus group 3, p < 0.001).

#### Hydropic placental pathologies group

Cases of non-molar hydropic abortion (group 4) and molar pregnancy (group 5) were included in this group.

Non-molar hydropic abortus group (group 4): The non-molar hydropic abortus group showed podoplanin staining of weaker diffuseness and intensity compared to the control group (group 1, *first trimester*). The non-molar hydropic abortus group had a mean podoplanin immunoreactivity score of 66.7241, compared to 69.6552 for the control group. However, this difference did not reach statistical significance (p = 1.00).

Molar pregnancy group (group 5): In tissues from partial molar pregnancies, podoplanin expression in the villi was heterogeneous. In edematous villi, expression was weak, whereas in fibrotic villi podoplanin positivity was stronger than in hydropic villi. In cistern-containing villi, podoplanin immunoreactivity was more pronounced in the stromal cells surrounding the cisterns than in other areas of the villous stroma. In addition, in some molar villi, podoplanin expression was concentrated in the sub-trophoblastic area. Podoplanin staining was homogeneous and weak in tissue samples from complete molar pregnancies (Fig. 1g–i). The mean podoplanin immunoreactivity score in the molar pregnancy group was 61.7241, significantly lower than that of the control group (group 1 versus group 5, p = 0.015).

#### Inflammation group

This group comprised third trimester placental tissues with acute placental inflammation (group 6). The podoplanin immunoreactivity of CVS cells was similar to that of the control group (group 3). Additionally, podoplanin immunoreactivity was not correlated with the localization or severity of placental inflammation (chorioamnionitis and/or funisitis and/or chorionic vasculitis). The mean podoplanin immunoreactivity score was 213.0000 in the acute placental inflammation group and 255.8824 in the control group. This difference was statistically non-significant (Fig. 1j–k, group 6 versus group 3, p = 0.47).

#### Hypoxia- ischemia group

Cases of IUGR (group 7) and preeclampsia (group 8), in which ischemic-hypoxic placental changes are predominant, were included in this group.

**Figure 2:**
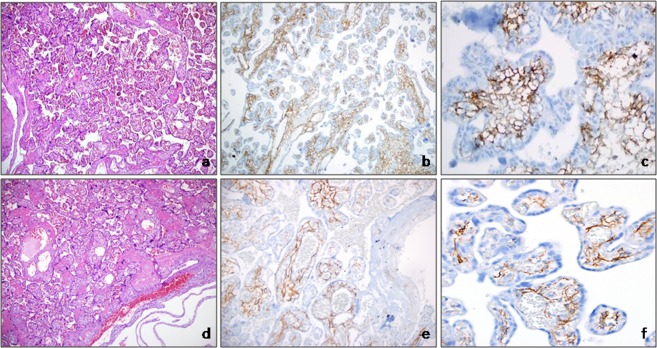
Group 8: In cases of preeclampsia, widespread and potent podoplanin immunoreactivity was observed in all types of villus (**a**, H&E, ×100; **b,c**, podoplanin, **b** ×100, **c** ×400). Group 7: In cases of IUGR, potent podoplanin immunoreactivity was observed in villous stroma (**d**, H&E, ×100; **e,f**, podoplanin, **e** ×100, **f** ×400).

In groups 7 and 8, podoplanin immunoreactivity in CVS cells increased homogeneously at all levels of the villous tree, from stem villi to distal villi. Podoplanin immunoreactivity was more pronounced in small villi, indicative of hypermaturation in the placenta. In cases of IUGR and preeclampsia, podoplanin immunoreactivity was strong in areas where reticular stroma could be observed in chorionic villi. However, podoplanin immunoreactivity was lost in fibrotic areas. In the CVS cells of the preeclampsia group, podoplanin immunoreactivity was more homogeneous and stronger than in all other groups, especially in the distal villi. Podoplanin immunoreactivity was significantly higher in the CVS cells of IUGR (group 7) and preeclampsia (group 8) tissues than in the third-trimester (group 3) control group (Fig. 2, group 7 versus group 3, p < 0.001; group 8 versus group 3, p < 0.001).

#### Podoplanin immunoreactivity gestational endometrium and other components of the placenta

**Figure 3:**
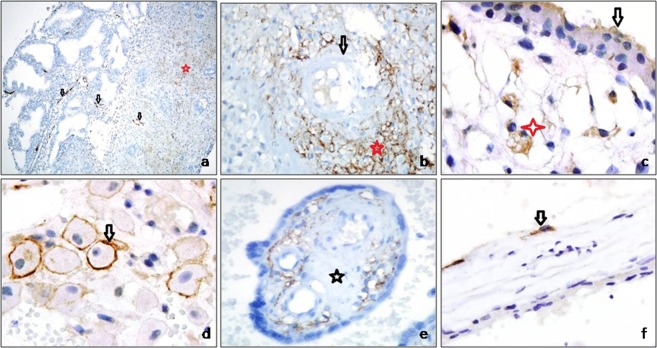
(**a**) In non-decidualized endometrium, the stroma is podoplanin-negative and contains many lymphatic vessels (arrow). Lymphatic regression is prominent in decidualized endometrium (asterisk indicates lymphatic vessels) (podoplanin, **a** ×50). (**b**) Strong expression of podoplanin in decidual cells around spiral arterioles (arrow, spiral arteriole; asterisk, podoplanin-positive decidual cell) (podoplanin, ×400). (**c**) Cytoplasmic podoplanin immunoreactivity in Hofbauer cells (asterisk) and apical immunoreactivity in syncytiotrophoblasts (arrow) (podoplanin, ×200). (**d**) Membranous podoplanin immunoreactivity in decidual cells (arrow) (podoplanin, ×100). (**e**) Loss of podoplanin expression in areas of stromal fibrosis (asterisks) in chorionic villi (podoplanin, ×200). (**f**) Podoplanin-positive stromal cells laying cisterns in molar villi (arrow) (podoplanin, ×630).

**Figure 4:**
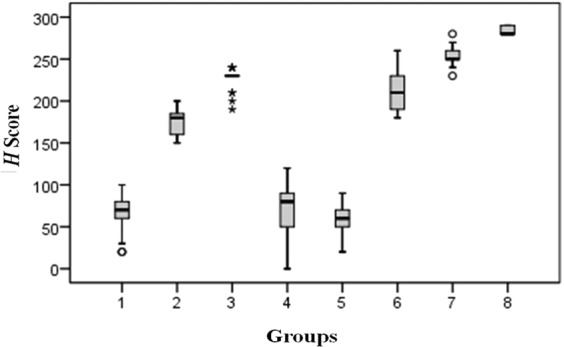
Box plot of H scores* [*Group 1 (first trimester, ≤13 weeks of gestation), group 2 (second trimester, 14–27 weeks), group 3 (term placenta, 28–40 weeks), group 4 (non-molar hydropic abortion), group 5 (molar pregnancy), group 6 (acute placental inflammation), group 7 (intrauterine growth retardation [IUGR]), and group 8 (preeclampsia)].

Podoplanin immunoreactivity was not detected in endometrial gland epithelial cells or non-decidualized stromal cells of the endometrium, but it was apparent in decidualized endometrial stroma. In decidualized endometrium, podoplanin immunoreactivity was seen in the cytoplasm and on the cell membrane, and was more prominent around spiral arterioles than in other areas. Very rarely, podoplanin immunoreactivity was observed in Hofbauer cells and in an apical-luminal reaction in syncytiotrophoblastic cells. Fetal membranes were negative for podoplanin (Figs 3, 4 and Table 2).

**Table 2:** Results of the statistical analyses*.

*H Score*	Group 1	Group 2	Group 3	Group 4	Group 5	Group 6	Group 7	Group 8
Median	70,0000	180,0000	230,0000	80,0000	60,0000	210,0000	250,0000	280,0000
Minimum	20,00	150,00	190,00	,00	20,00	180,00	230,00	280,00
Maximum	100,00	200,00	240,00	120,00	90,00	260,00	280,00	290,00
Mean	66,7241	176,0000	215,8824	69,6552	61,7241	213,0000	254,3333	284,0000
Std. dev.	18,67430	17,88854	14,60258	31,45095	15,59967	27,10064	12,22866	5,47723
	(χ^2^ = 157.073; p < 0.001).							
Group 1	—	<0.001	<0.001	1.00	0.015	0.001	<0.001	<0.001
Group 2	<0.001	—	<0.001	0.19	0.001	0.001	<0.001	<0.001
Group 3	<0.001	<0.001	—	<0.001	<0.001	0.47	<0.001	<0.001
Group 4	1.00	0.19	<0.001	—	0.019	<0.001	<0.001	<0.001
Group 5	0.015	0.001	<0.001	0.019	—	<0.001	<0.001	<0.001
Group 6	<0.001	<0.001	0.47	<0.001	<0.001	—	<0.001	<0.001
Group 7	<0.001	<0.001	<0.001	<0.001	<0.001	<0.001	—	0.002
Group 8	<0.001	<0.001	<0.001	<0.001	<0.001	<0.001	0.002	—

^*^Group 1 (first trimester, ≤13 weeks of gestation), group 2 (second trimester, 14–27 weeks), group 3 (term placenta, 28–40 weeks), group 4 (non-molar hydropic abortion), group 5 (molar pregnancy), group 6 (acute placental inflammation), group 7 (intrauterine growth retardation, IUGR), and group 8 (preeclampsia).

## Discussion

In parallel with fetal growth, placental tissue must adapt both physically and functionally to maintain a healthy pregnancy. The most important adaptation mechanism is the initiation and maturation of fetal angiogenesis in chorionic villi. In most regions of the body, angiogenesis parallels lymphangiogenesis. Castro and Lundell^10,17^ reported that lymphatic vessels regress in the gestational endometrium. This suggests that lymphangiogenesis in placental tissue exhibits a pattern different from angiogenesis.

We detected a large number of well-developed, open lumen lymphatic vessels in the non-decidualized gestational endometrium. However, the number of lymphatic vessels decreased with the onset of decidualization, and the lymphatic vessels collapsed or narrowed. Also, the decidua did not contain lymphatic vessels. These findings indicate that endometrial lymphatics regress during the gestational period, as opposed to the development of blood vessels. The eradication of a mature lymphatic system suitable for adult life in the gestational endometrium may contribute to the creation of an immunologically safe pregnancy environment, promoting feto-maternal tolerance^6–8,11,13,17^.

Stromal cells in the non-decidualized endometrium were negative for podoplanin, whereas those of the decidualized endometrium were podoplanin-positive. Lundell *et al*.^17^ reported that B cell activating factor production by podoplanin-positive decidual stromal cells could be important for local B cell homeostasis during pregnancy. In our study, the higher level of podoplanin expression in decidual cells located near inflammatory cells supports the hypothesis of related to the immunomodulatory function of decidual cells.

Another region of the gestational endometrium characterized by prominent podoplanin expression was the decidual cells around spiral arterioles. Volchek *et al*.^11^ reported that lymphatic regression in the gestational endometrium begins around the spiral arterioles, and that decidualized cells in the vicinity of these vessels show strong podoplanin positivity. The authors proposed that podoplanin released from regressed lymphatic vessels is phagocytosed by periarteriolar decidual cells. In our study, podoplanin expression in those cells was more intense than in other regions. Podoplanin may have important effects on pericyte migration^25^, our findings suggest that periarteriolar podoplanin-positive decidual cells play a role in vascular maturation and function.

Podoplanin is a transmembrane protein. A membranous reaction is evident in epithelial cells, such as those of mesothelioma, whereas cytoplasmic staining is dominant in spindle cells, such as follicular dendritic cells^5,7,9^. In our study, membranous staining was detected in decidual cells with an epithelioid appearance, and cytoplasmic staining in CVS cells.

The lymphatic system is involved in the regulation of interstitial fluid homeostasis and immunogenicity and is present throughout the body, except in immunologically conserved sites, such as the placenta, brain, eye, and testes^5,25–29^. Podoplanin is a protein expressed by lymphatic endothelium and regulated by the lymphatic-specific homeobox gene Prox1^1,4^. Podoplanin is important in lymphangiogenesis as well as angiogenesis. Inhibition of podoplanin disrupts angiogenesis and results in the formation of blood vessels that are convoluted, devoid of adequate pericyte support, permeable, and susceptible to bleeding^25^.

The CLEC-2 receptor is expressed mainly on megakaryocytes, platelets, and leukocytes, and its only known endogenous ligand is podoplanin. Podoplanin, which forms a heteroligand with CLEC-2, activates the ERM complex in podoplanin receptor-bearing cells (e.g., endothelial cells, follicular dendritic cells, and activated fibroblasts) and actin-dependent remodeling of the cytoskeleton. The CLEC-2/podoplanin interaction accelerates the chemotaxis and migration of endothelial cells, induces platelet activation-aggregation, and affects the coagulation system^2,4,25,30^. In animal experiments, the interaction of CLEC-2/podoplanin was inhibited using an anti-podoplanin antibody. Thus, in patients with sepsis and disseminated intravascular coagulation, in which there is excessive endothelial activation and vascular permeability, antipodoplanin antibody administration may be an effective form of treatment^4,23,30,31^.

Preeclampsia is a multisystemic disease specific characterized by maternal de-novo hypertension, proteinuria and, on a cellular level, endothelial activation, a hyperinflammatory response, and thrombus formation^16,23^. Abnormal placentation during the early stages of pregnancy causes an uncontrolled molecular transition from the intervillous space to the maternal circulation, resulting in preeclampsia. While the mechanism has yet to be fully elucidated, both abnormal trophoblast invasion and inadequate uterine spiral artery remodeling have been implicated in preeclampsia^31,32^.

Podoplanin expression in preeclampsia has been the subject of several studies. He *et al*.^16^ investigated gene expression profiles in normal (n = 8) and preeclamptic placentas (n = 8), and showed that the podoplanin gene is down-regulated in preeclamptic placental tissue. Wang *et al*.^7^ used immunohistochemistry and western blotting to show a decrease in podoplanin levels in the placentas of preeclamptic patients (n = 5); this was implicated in defective interstitial fluid homeostasis and trophoblast invasion, and therefore possibly in the etiology of preeclampsia. Hayashi *et al*.^32^ examined first-trimester placental cell lines and showed that podoplanin and podoplanin-activated RhoA protein increased in placental tissue under hypoxic conditions. In the study of Zhou *et al*.^31^ the levels of podoplanin-activated RhoA and HIF-1 protein increased significantly in preeclamptic placentas. Hypoxia also causes an increase in podoplanin expression in stromal cells of the eye and brain, both of which, like the placenta, lack a mature lymphatic system^25–28^.

In our study, podoplanin expression in CVS cells was significantly higher in the placentas of preeclamptic patients than in those of the normotensive control group. A similar result was obtained in the IUGR group with uteroplacental insufficiency. Our findings suggest that podoplanin expression increases in placental CVS cells under hypoxic-ischemic conditions. In addition to supporting previously published data, the results of this study suggest that the ischemic response of placental tissue at least in part develops through podoplanin, which then leads to the activation of Rho family proteins as well as the HIF-1 and ERM complexes.

In addition, our results suggest that high-level podoplanin expression in CVS cells contributes to the development of clinical symptoms of preeclampsia. The CLEC-2/ podoplanin interaction induces endothelial activation, platelet activation/aggregation, and inflammation. These effects play a role in the pathogenesis of preeclampsia^14,23,30,32^. Therefore, the development of pharmacological agents that modulate the activity of podoplanin would provide a new option for the treatment of preeclampsia.

Hydatiform mole is a trophoblastic disease of androgenic origin that is characterized by abnormal hyperproliferative trophoblastic activity and hydropic degeneration in placental villi. Despite different pathogenetic mechanisms, the morphological appearance of the various forms of hydatiform mole are similar. Both an increase in apoptosis in endothelial precursors and inadequate pericytic migration in villous stromal vessels have been implicated in the etiology of the disease. The deficiency in vascular maturation is thought to induce villous swelling, which is an important morphological finding of molar pregnancy^10,12,15^.

The expression of podoplanin in molar placentas has been investigated in only two published studies. Heazel *et al*.^15^ reported podoplanin immunoreactivity in the CVS cells of placental mesenchymal dysplasia, the etiology of which was suggested to involve abnormal lymphangiogenesis. Castro *et al*.^10^ investigated prox1 and podoplanin expression as markers of lymphatic differentiation in four molar pregnancies and two spontaneous abortions. They showed that CVS cells in normal and molar placentas are positive for podoplanin but do not express prox-1, and that cells within the cisterns of molar placentas were stromal, not endothelial cells.

Here, we showed that podoplanin expression was lower in villi with molar changes than in control tissues, and that the severity of hydropic degeneration correlated negatively with the decrease in podoplanin expression. Podoplanin is important in interstitial fluid homeostasis, and podoplanin-positive stromal cells in uveoscleral tissue were shown to play a role in interstitial fluid transport, by forming lymphatic-like channels^26,27^. In animal experiments, a mutation in the podoplanin gene resulted in widespread lymphedema^29^. Our findings support the view that inadequate podoplanin activity leads to an impaired interstitial fluid balance in placental tissue. The decrease in podoplanin function may thus contribute to the formation of edematous villi in molar pregnancy. Additionally, defective vascularization of chorionic villi is the most important mechanism and morphological finding in the pathogenesis of molar pregnancy^22^. Podoplanin plays a role not only in lymphangiogenesis but also in angiogenesis and vascular maturation. The absence of, or an inadequacy in, podoplanin during embryogenesis or in adult brain/eye tissues causes a defect in the initiation, development, and maturation of angiogenesis^25^. Our results suggest that a similar mechanism is responsible for molar pregnancy. An inadequate level of podoplanin in molar placental tissue may prevent the formation of fetal vessels or cause them to regress through abnormal endothelial apoptosis.

We examined control placental tissues for podoplanin expression in different gestational periods. The lowest levels of expression were in CVS cells in the early gestational period, before fetal blood vessels had formed. Between the onset of fetal vascularization and the termination of pregnancy, podoplanin expression by CVSs gradually increases. Our study, the only detailed investigation of placental maturation and podoplanin expression performed thus far, suggests that the increase in podoplanin in CVS cells during pregnancy is related to the healthy development and continuation of fetal vascularization.

In summary, podoplanin is a cell-surface receptor whose effects include cell adhesion, chemotaxis, and lymphatic and vascular maturation. It is expressed in human placenta, mainly by CVS, and in decidual cells. Podoplanin plays an important role in physiological and pathological processes in the placenta, and in non-placental tissues. Our results implicate podoplanin expression in CVS cells in two important placental pathologies, preeclampsia and hydatiform mole, with expression of the protein differing significantly in these tissues compared to normal placental tissues at the same gestational week. In the preeclamptic placenta, podoplanin expression by CVS cells may increase in response to hypoxic-ischemic conditions, whereas in molar pregnancies, decreased expression may account for the observed villous swelling, by interrupting intercellular fluid homeostasis. Molecular studies in a larger number of tissue samples will shed further light on the biological importance of podoplanin in placental tissue.

## References

[1] Ugorski M, Dziegiel P, Suchanski J (2016). Podoplanin - a small glycoprotein with many faces. Am. J. Cancer Res..

[2] Quintanilla Miguel, Montero-Montero Lucía, Renart Jaime, Martín-Villar Ester (2019). Podoplanin in Inflammation and Cancer. International Journal of Molecular Sciences.

[3] Krishnan H, Miller WT, Blanco FJ, Goldberg GS (2019). Src and podoplanin forge a path to destruction. Drug Discov. Today..

[4] Krishnan H (2018). Podoplanin - an emerging cancer biomarker and therapeutic target. Cancer. Sci..

[5] Dobbs LG, Williams MC, Gonzalez R (1998). Monoclonal antibodies specific to apical surfaces of rat alveolar type I cells bind to surfaces of cultured, but not freshly isolated, type II cells. Biochim. Biophys. Acta..

[6] Bellini C (2012). Are there lymphatic vessels in the placenta?. Lymphology..

[7] Wang Y (2011). D2-40/podoplanin expression in the human placenta. Placenta..

[8] Liu JL, Zuo RJ, Peng Y, Fu YS (2016). The Impact of Multiparity on Uterine Gene Expression and Decidualization in Mice. Reprod. Sci..

[9] Lee BS (2014). Primo vascular system in human umbilical cord and placenta. J. Acupunct. Meridian. Stud..

[10] Castro E, Tony Parks W, Galambos C (2011). Neither normal nor diseased placentas contain lymphatic vessels. Placenta..

[11] Volchek M (2010). Lymphatics in the human endometrium disappear during decidualization. Hum. Reprod..

[12] Bellini C (2010). Immunohistochemistry in non-immune hydrops fetalis: a single center experience in 79 fetuses. Am. J. Med. Genet. A..

[13] Rogers PA, Donoghue JF, Girling JE (2008). Endometrial lymphangiogensis. Placenta..

[14] Onyangunga O, Moodley J, Odun-Ayo F, Naicker T (2018). Immunohistochemical localization of podoplanin in the placentas of HIV-positive preeclamptic women. Turk J Med Sci..

[15] Heazell AE, Sahasrabudhe N, Grossmith AK, Martindale AE, Bhatia KA (2009). A case of intrauterine growth restriction in association with placental mesenchymal dysplasia with abnormal placental lymphatic development. Placenta..

[16] He P, Shao D, Ye M, Zhang G (2015). Analysis of gene expression identifies candidate markers and pathways in pre-eclampsia. J. Obstet. Gynaecol..

[17] Lundell, A. *et al*. IFN type I and II induce BAFF secretion from human decidual stromal cells. *Sci. Rep*. **6**, 10.1038/srep39904 (2017). 10.1038/srep39904 PMC5216379 28057926

[18] Vogler C, Petterchak J, Sotelo-Avila C, Thorpe C (2000). Placental pathology for the surgical pathologist. Adv. Anat. Pathol..

[19] Banet N (2014). Characteristics of hydatidiform moles: analysis of a prospective series with p57 immunohistochemistry and molecular genotyping. Mod. Pathol..

[20] Kim, C. *et al*. Acute chorioamnionitis and funisitis: definition, pathologic features, and clinical significance. *Am. J. Obstet. Gynecol*. **2015**: 29–52 (213). 10.1016/j.ajog.2015.08.040 PMC4774647 26428501

[21] Nardozza LM (2017). Fetal growth restriction: current knowledge. Arch. Gyneco. l Obstet..

[22] Howat AJ (1993). Can histopathologists reliably diagnose molar pregnancy?. J. Clin. Pathol..

[23] Duhig K, Vandermolen B, Shennan A (2018). Recent advances in the diagnosis and management of pre-eclampsia. F1000Res..

[24] Ozbilgin, K. *et al*. The expression of Forkhead transcription factors in decidua and placenta in patients with missed abortion. *Clin. Exp. Obstet. Gynecol*. 42510–514 (2015). 26411222

[25] Lowe KL (2015). Podoplanin and CLEC-2 drive cerebrovascular patterning and integrity during development. Blood..

[26] Loukovaara Sirpa, Gucciardo Erika, Repo Pauliina, Lohi Jouko, Salven Petri, Lehti Kaisa (2015). A Case of Abnormal Lymphatic-Like Differentiation and Endothelial Progenitor Cell Activation in Neovascularization Associated with Hemi-Retinal Vein Occlusion. Case Reports in Ophthalmology.

[27] Loukovaara S (2015). Indications of lymphatic endothelial differentiation and endothelial progenitor cell activation in the pathology of proliferative diabetic retinopathy. Acta Ophthalmol..

[28] Kolar K (2015). Podoplanin: a marker for reactive gliosis in gliomas and brain injury. J. Neuropathol. Exp. Neurol..

[29] Schacht V (2003). T1alpha/podoplanin deficiency disrupts normal lymphatic vasculature formation and causes lymphedema. EMBO. J..

[30] Suzuki-Inoue K, Osada M, Ozaki Y (2017). Physiologic and pathophysiologic roles of interaction between C-type lectin-like receptor 2 and podoplanin: partners from in utero to adulthood. J. Thromb. Haemost..

[31] Zhou L, Qiao F (2006). Expression of RhoA in placenta of preeclampsia. J. Huazhong. Univ. Sci. Technolog. Med. Sci..

[32] Hayashi M (2005). Hypoxia up-regulates hypoxia-inducible factor-1alpha expression through RhoA activation in trophoblast cells. J. Clin. Endocrinol. Metab..

